# Posterior-only Stabilization for Traumatic Thoracolumbar Burst Fractures

**DOI:** 10.7759/cureus.2296

**Published:** 2018-03-09

**Authors:** Omid R Hariri, Samir Kashyap, Ariel Takayanagi, Chris Elia, Quang Ma, Dan E Miulli

**Affiliations:** 1 Department of Neurosurgery, Stanford University School of Medicine; 2 Department of Neurosurgery, Riverside University Health System Medical Center, Moreno Valley, California, United States; 3 Department of Neurosurgery, Neurospine Institute, Palmdale, Ca

**Keywords:** operative technique, thoracolumbar, burst fractures, posterior stabilization, spinal fusion, trauma, spine, pedicle screw fixation

## Abstract

Background

No consensus exists for the management of unstable thoracolumbar (TL) burst fractures. Surgical options include anterior, lateral, or posterior stabilization (or a combination), depending on the fracture. The potential benefits of anterior reconstruction come with increased operative time and associated morbidity. A posterior-only approach can offer stable correction without increased operative risks but may result in loss of kyphotic correction over time.

Purpose

To determine whether posterior-only stabilization is a viable treatment option for patients with traumatic TL fractures as opposed to anterior and combined approaches.

Methods

We performed a retrospective analysis of adult patients with TL burst fractures who underwent posterior­-only surgical intervention from 2005 to 2015. Operations were performed at two levels above and below the fractured segment using pedicle screw-rod fixation constructs with autograft and allograft. All patients received TL bracing for at least three months. Patients lost to follow­up were excluded.

Results

Sixty-four consecutive patients with posterior­-only stabilization were identified, with 18 lost to follow­up. Of the remaining 46 patients, 93% (n=43) were male and 7% (n=3) were female, with a mean age of 36.8 years. All patients were followed for 12 months. The mean time until the removal of the brace was 3.54 months. No patients required additional surgical intervention for spinal stabilization. Three patients experienced postoperative complications, all of which were related to infection.

Conclusions

Our data indicate that posterior­-only stabilization for traumatic TL burst fractures is a durable and effective option in select patients. The approach offers surgical intervention with a decreased perioperative risk as well as reduced morbidity and mortality, with a minimal increase in the risk of kyphotic deformity. Further prospective studies are necessary to validate these findings clinically.

## Introduction

The optimal management of thoracolumbar (TL) fractures continues to be debated. While most patients are managed conservatively, surgery is indicated in patients whose the fracture mechanism and acquired traumatic deformity is deemed unstable. The goals of surgery are to fuse the fewest number of segments needed to obtain a well-balanced spine with lasting stability while reducing the risk of complications associated with large-scale operations. There continues to be a lack of consensus on the appropriate surgical approach with regard to these objectives.

A large systematic review showed that combined approaches result in a slightly higher rate of kyphotic correction. Mean kyphotic correction was 3.42 degrees greater with the combined approaches (p<.00001) [1]. In cases of severe canal compromise, anterior approaches, which lead to direct decompression, may be necessary [2]. While anterior approaches may be superior for the correction of deformity, they are associated with longer operating times and increased blood loss [3]. Given that most patients with traumatic injuries of the spine have other associated injuries, the risks must be carefully weighed against the benefits when considering surgical management of TL instability.

Anterior column support usually entails the placement of expandable cages or strut grafts via cavitary or extracavitary access in addition to a posterior construct. Typical posterior fixation techniques consist of titanium transpedicular distraction and screw fixation with rods and graft placement bilaterally.

Numerous studies have shown that the posterior-only management of TL burst fractures is an effective alternative. Been et al. showed that a posterior-only approach produced kyphotic correction, the stability of correction, and neurological status results similar to those of the combined anterior and posterior management of TL burst fractures [4]. Similarly, Inamasu et al. reported that posterior-only fixation was associated with effective stabilization and low complication rates [5]. Here, we present a single institution's experience with consecutive TL burst fracture patients who underwent a posterior-only stabilization over a period of 10 years to determine whether posterior-only stabilization is a viable treatment option for patients with traumatic TL fractures as opposed to the anterior and combined approaches.

## Materials and methods

Data were collected from a retrospective review of a prospectively collected database. We selected consecutive TL burst fracture patients treated via a surgical procedure who presented to our institution from 2005 to 2015. No anterior approaches were performed. The year 2005 marked the earliest date the electronic medical record was implemented at our institution. Chance type, flexion distraction, and degenerative and pathological fractures were excluded. The department of neurosurgery performed all operations included in this study. The surgical management of these patients consisted of two levels above and below the fractured segment, with pedicle screw-rod fixation constructs along with autograft and allograft. Decompressive laminectomy was performed at the index level. No additional biological fusion materials were utilized. All patients were treated in the same hospital stay with follow-up assessments upon discharge in the neurosurgical clinic. According to our practice, all patients were treated with TL bracing when out of bed for a minimum of three months or until patients were pain-free, with stable TL flexion-extension x-rays.

Inclusion criteria

From 2005 to 2015, all consecutive adult patients (>18 years old) with traumatic TL burst fractures managed operatively were selected. Data were collected, including age, sex, neurological status, time to removal of the brace with stable flexion/extension plain x-rays, and need for additional surgery. To be included in our study, patients needed a minimum of three months postoperative follow-up evaluations. Following the documentation of stable flexion/extension and the removal of bracing, patients were discharged from the neurosurgery clinic with appointments as needed. The failure of the initial posterior surgery was defined as any patient who required additional operative spinal stabilization either for neurological or mechanical instability during the postoperative years in which we followed the patient.

Exclusion criteria

Patients were considered lost to follow-up if they did not present to the neurosurgery clinic upon discharge. Degenerative or pathological indications for surgery were also excluded.

## Results

A total of 64 consecutive patients were identified who underwent surgical management of unstable TL injuries from 2005 through 2015. Of the 64 patients, 18 were lost to follow-up. Forty-six patients (n = 46; 43 men, 3 women) had adequate postoperative follow-up data for review. Of the patients with adequate follow-up, the mean age was 36.8 years and the mean time until stable flexion-extension x-rays and removal of the brace was 3.54 months (Table 1).

**Table 1:** Summary of results

Patient Characteristics			
Total number of patients			64
	Lost to follow-up		28% (n=18)
	Included patients		72% (n=46)
Female			7% (n=3)
Male			93% (n=43)
Mean age (years)			36.8
Patients requiring additional stabilization after initial operation			0
Mean time until stable flexion-extension x-rays (months)			3.54

Of all patients seen postoperatively, none required additional surgical intervention for spinal stabilization. All patients included in this study were followed for 12 months. In terms of postoperative complications, the development of osteomyelitis from an adjacent sacral decubitus ulcer occurred in one patient. This patient’s neurological status was complete paraplegia, ventilated with poorly compensated congestive heart failure. Two separate patients also developed wound dehiscence not requiring additional surgical procedures other than washout and primary reclosure (Table 2).

**Table 2:** Postoperative complications *From adjacent sacral decubitis ulcer **Both patients required washout and closure but no other surgeries.

Complication		Number of Patients	% Overall
Total		3	6%
Osteomyelitis*		1	2%
Wound dehiscence**		2	4%

**Figure 1:**
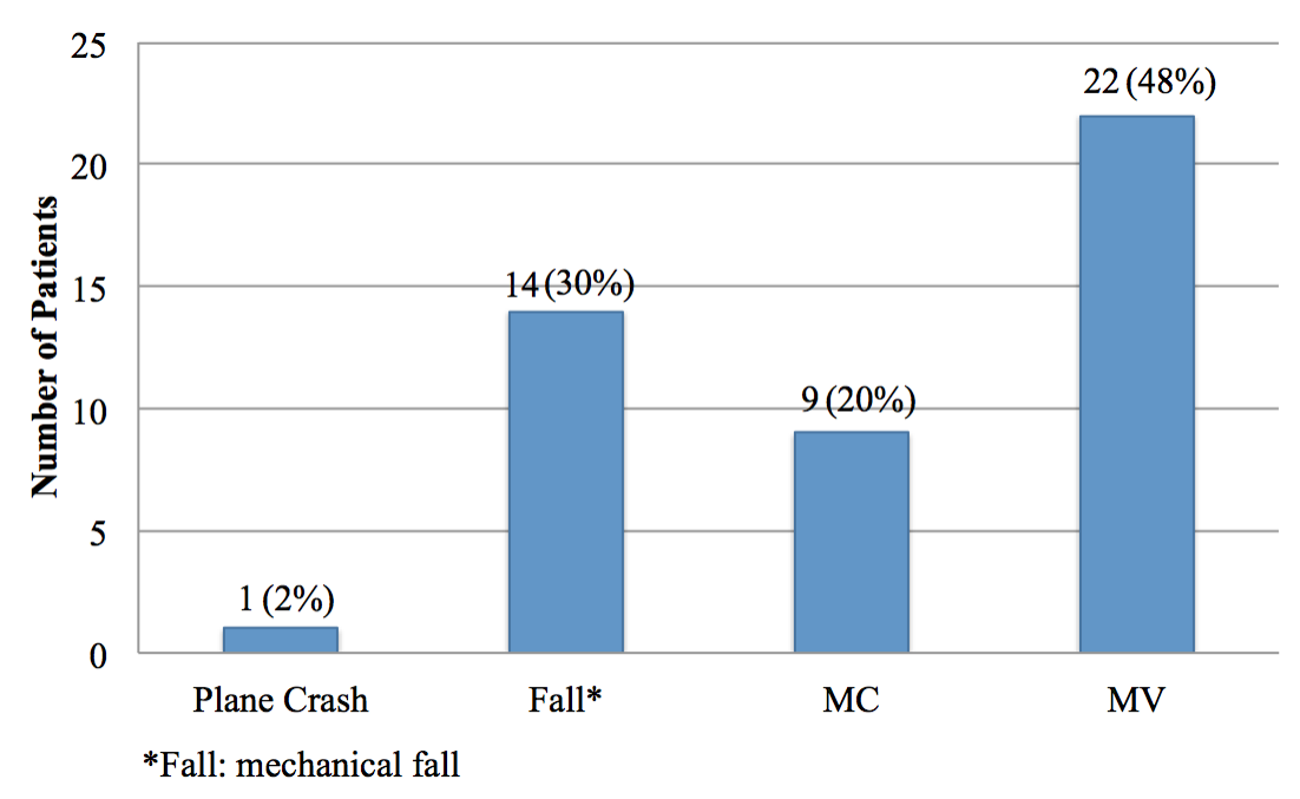
Mechanism of injury Abbreviations: MC, motorcycle; MV, motor vehicle.

**Figure 2:**
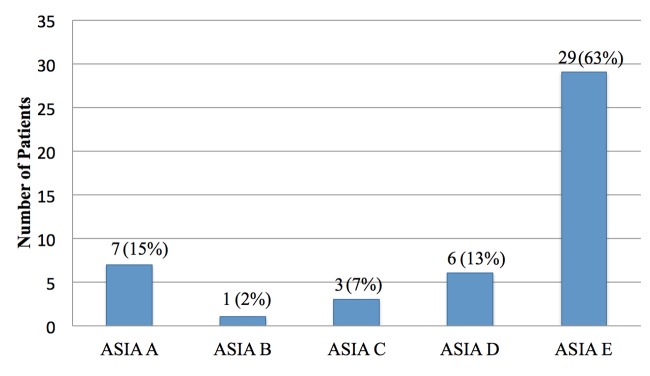
ASIA impairment scale Abbreviation: ASIA, American Spinal Injury Association.

**Figure 3:**
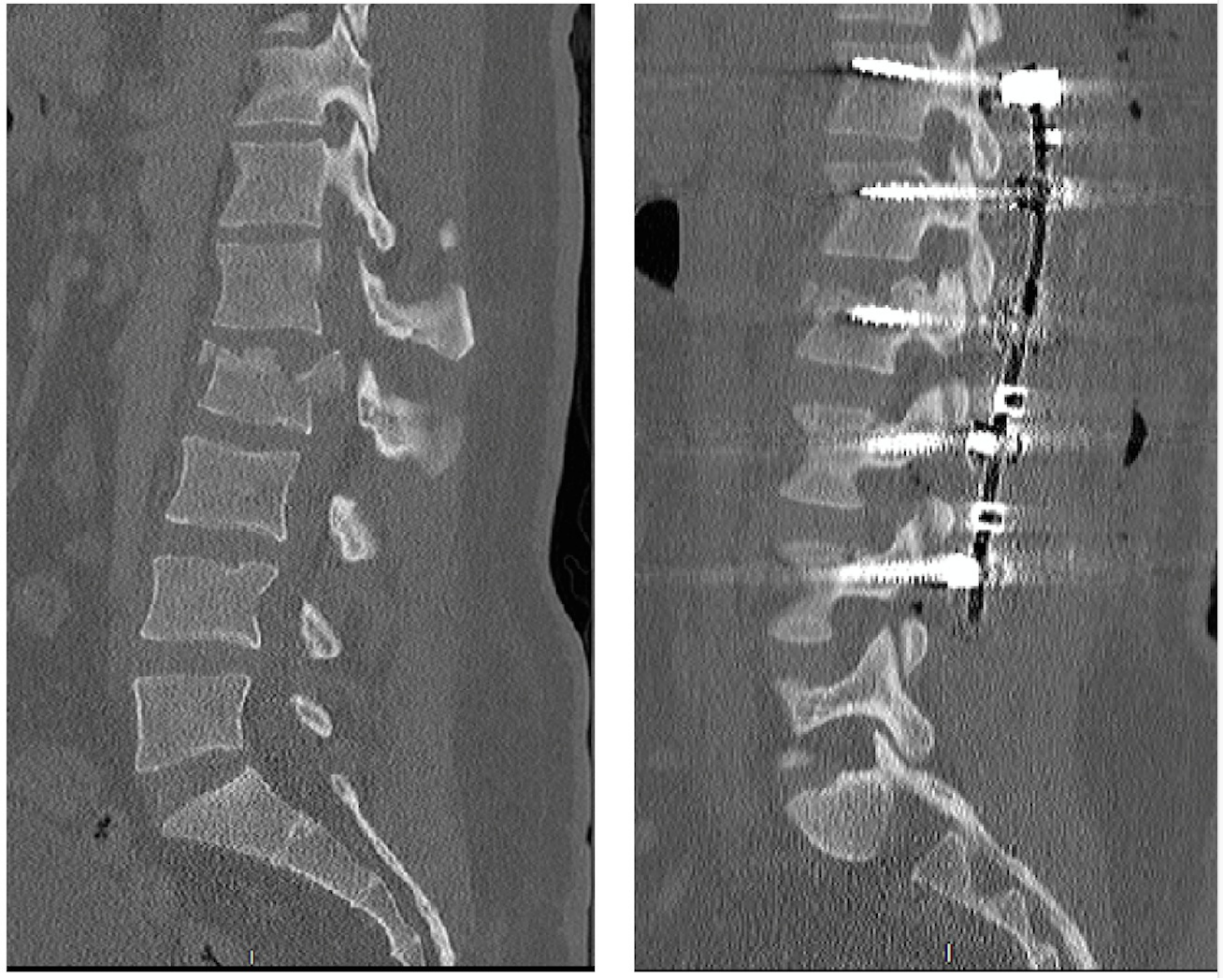
Typical preoperative and postoperative imaging for a patient included in this study Typical preoperative (left) and postoperative (right) computed tomography scan of thoracolumbar (TL) spine in a patient with traumatic L2 burst fracture with 30% retropulsion, 10% angulation, and 75% of the spinal canal is demonstrated. Hardware shown to illustrate the typical posterior-only approach used in this study.

The most common mechanisms of injury were injuries from motor vehicle and motorcycle crashes, accounting for 67% (n=31) of the injuries sustained in our study (Figure 1). The majority of our patients did not exhibit any neurologic deficit per the American Spinal Injury Association impairment scale (Figure 2). Figure 3 illustrates preoperative and postoperative imaging for a typical patient included in this study.

## Discussion

TL fractures are a common form of spinal trauma, comprising 15% to 20% of traumatic spinal fractures, with an overall incidence of 64 per 100,000 people per year [6-7]. A majority occur secondary to high-energy trauma in younger patients, while in older patients, the more common etiology is ground-level trauma to an osteoporotic spine [8].

The goals of surgery should be tailored to each patient’s needs and based on the goal of achieving a well-balanced, dynamically stable, painless, durable spine with the fewest number of fused segments. This must be done while reducing the risk of the complications associated with large-scale operations and obtained traumatic injuries.

The traditional argument has been centered on whether to stabilize with the re-establishment of the anterior column with anterior approaches, by posterior-only approaches, or by a combination of the two. A contemporary approach to vertebral body height restoration via balloon vertebroplasty or kyphoplasty in combination with posterior fixation has been slowly gaining popularity, with some studies reporting good success with this approach. In addition to traumatic fractures, cement augmentation combined with posterior fusion has also been used successfully in the setting of pathological fractures [9-12]. Anterior approaches allow access to ventral bone fragments, leading to direct decompression. This is in contrast to posterior approaches, which typically achieve decompression indirectly via ligamentotaxis and realignment of bone fragments [4].

A retrospective study of 46 patients with traumatic TL burst fractures, which compared approaches, showed that while a loss of reduction greater than five degrees occurred more often in the posterior-only group (68% versus 7%), neurological improvement occurred regardless of approach. In addition, a loss of correction was not associated with increased pain in the posterior-only group [4].

The Spine Study Group of the German Association of Trauma Surgery performed a prospective multicenter study of 448 traumatic TL fractures treated with posterior-only approaches versus 197 treated with a combined approach. Combined approaches achieved a significantly larger kyphotic correction with a reduction of Cobb’s angles by 10.4 degrees and 13.8 degrees, respectively (p<0.01). Similarly, the correction of vertebral body height (sagittal index) was significantly greater in the combined group (0.3) versus the posterior-only group (0.2) [13-15]. These studies suggest that post-traumatic deformity can best be corrected with combined approaches compared to posterior-only approaches. While combined approaches may be superior in this aspect, the patient’s other traumatic injuries and overall status must be considered.

The posterior-only approaches have been shown to have significantly reduced operative time (219 minutes) compared to the combined approaches (569 minutes, p<0.0003) [1]. Blood loss and packed red blood cell transfusions required were significantly less in posterior-only approaches (1103 mL, 2.3 units) compared to the anterior approaches (2541 cc, 4.3 units). Patients with traumatic unstable burst fractures, many of whom have multiple injuries, may benefit from the decreased operative time, blood loss, and transfusions, which can be achieved with the posterior-only approaches.

An updated multicenter study by the German Association of Trauma Surgery showed that although radiographic deformity was best addressed with a combined approach, patients treated with posterior-only approaches had better functional outcomes based on visual analog scale spine scores compared to those treated with combined approaches [16]. Other studies have shown no significant difference in return to work or quality of life between the two groups [1].

Our data suggest that in young males, a posterior-only surgical approach for traumatic TL fractures is an effective treatment. None of the patients in the present study who received posterior-only stabilization for traumatic TL burst fractures required additional surgical stabilization after a one-year follow-up. Unfortunately, given our follow-up status, we were not able to study the results of proximal junctional deformity or further need for corrective or revision surgery in two-year follow-ups.

The population in this study consisted primarily of young male patients. This population is associated with high fusion rates, and this may contribute to our observation that patients included in the study did not require anterior column support. While this is a limited demographic, two-thirds of traumatic TL burst fractures occur in male patients and most occur in young patients with a peak age of 20 to 40 years [8]. Therefore, our findings are applicable to many patients who endure traumatic TL fractures.

We recognize that the sample size and follow-up time of one year are limitations of our study. Future studies may be done to better compare the operative risk of approaches in traumatic TL fractures. Randomized controlled trials are necessary to further define the indications of various approaches for traumatic TL fractures.

## Conclusions

In conclusion, our results demonstrate that young male patients with traumatic TL fractures can be effectively managed with posterior-only surgical intervention; thus avoiding the inherent risks and costs associated with anterior column support. Our data parallel previously published reports that posterior stabilization is a long-lasting, effective approach to the treatment of traumatic TL burst fractures.
